# Blood Pressure and Surface Temperature in 110 Cases of Shell Shock

**Published:** 1917-11

**Authors:** 


**Blood Pressure and Surface Temperature in 110 Cases of Shell Shock.** By Edith M. N. Green, Assistant to Maj. F. W. Mott at Maudsley Hospital. Abstracted from the *Lancet*, Sept. 22, 1917.

Of 110 shell shock cases, 55 had pressure below 120 mm. Hg. 25 were between 88 and 110. Of the less severe cases 28 were between 130 and 150, and 27 between 120 and 130. Of those with a pressure above 130 only 4 were severe cases.

Symptoms accompanying low pressure were: terrifying dreams with sweating and trembling, dusky, clammy hands with a tremor, fatiguability and irritability, depression, lack of self-confidence and initiative, headache, and, at time of admission, dilated pupils.

Only 10 of the men with a pressure above 120 suffered from nightmare. These only slightly. In all cases, however, headache was a common symptom.

Usually the variation in blood pressure and surface temperature corresponded to changes in the general condition and symptoms of the patient. Falls in blood pressure and surface temperature did not, however, always coincide. No cases showed organic lesion and in all cases the urine was normal.

The somewhat permanent vaso-motor disturbance produced at the time of the shock, as indicated by the constant relationship between low blood pressure and terrifying dreams, was aggravated by the cerebral anemia resulting from the low blood pressure, which lessened mental and physical activity and rendered the patients less able to throw off the bad dreams. Rise in the blood pressure was almost always accompanied by lessening of the terror.

Pituitrin Treatment: given (1) as pituitary fluid 0.5 c. c. b. d. by injection; (2) as ext. pituitrin gr. ii. t.d.s. The extract proved more satisfactory since it was not accompanied by dizziness. Blood pressure rose immediately — in one case from 100 to 122 - and there followed a marked general improvement. When polyuria resulted the treatment was suspended until the urine became normal, but in no case did the blood pressure fall to the original level.

Thyroid Extract Treatment: Doses 1/4 to gr. i., t.d.s. Success in 13 out of 20 cases. When coupled with pituitrin, however, the effect was more rapid. The instrument used was Rogers's sphygmomanometer (Short and Mason, Ltd.).

